# Impact of different ventilation conditions on tobacco smoke-associated particulate matter emissions in a car cabin using the TAPaC platform

**DOI:** 10.1038/s41598-023-35208-2

**Published:** 2023-05-22

**Authors:** Lukas Pitten, Dörthe Brüggmann, Janis Dröge, Markus Braun, David A. Groneberg

**Affiliations:** Institute of Occupational Medicine, Social Medicine, and Environmental Medicine, Goethe University Frankfurt, Theodor-Stern-Kai 7, 60590 Frankfurt am Main, Germany

**Keywords:** Health care, Risk factors

## Abstract

Despite antagonizing attempts from the tobacco industry, passive inhalation of tobacco smoke is known to be cancerogenic and toxic to human health for decades. Nonetheless, millions of non-smoking adults and children are still victims of second-hand smoke. Accumulation of particulate matter (PM) in confined spaces such as the car are particularly harmful due to high concentrations. We here aimed to analyze the specific effects of ventilation conditions in the setting of a car. By the use of the measuring platform TAPaC (tobacco-associated particulate matter emissions inside a car cabin), 3R4F reference cigarettes, Marlboro red, and Marlboro gold were smoked in a car interior with a volume of 3.709 m^3^. Seven different ventilation conditions (C1–C7) were analyzed. Under C1, all windows were closed. Under C2–C7, the car ventilation was turned on power level 2/4 with the air directed towards the windshield. Only the passenger side window was opened, where an outer placed fan could create an airstream speed of 15.9–17.4 km/h at one meter distance to simulate a driving car. C2: Window 10 cm opened. C3: Window 10 cm opened with the fan turned on. C4: Window half-opened. C5: Window half-opened with the fan turned on. C6: Window fully opened. C7: Window fully opened with the fan turned on. Cigarettes were remotely smoked by an automatic environmental tobacco smoke emitter and a cigarette smoking device. Depending on the ventilation condition the cigarettes emitted different mean PM concentrations after 10 min under condition C1 (PM_10_: 1272–1697 µg/m^3^, PM_2.5_: 1253–1659 µg/m^3^, PM_1_: 964–1263 µg/m^3^) under C2, C4, and C6 (PM_10_: 68.7–196.2 µg/m^3^, PM_2.5_: 68.2–194.7 µg/m^3^, PM_1_: 66.1–183.8 µg/m^3^) C3, C5, and C7 (PM_10_: 73.7–139 µg/m^3^, PM_2.5_: 72–137.9 µg/m^3^, PM_1_:68.9–131.9 µg/m^3^). Vehicle ventilation is insufficient to protect passengers from toxic second-hand smoke completely. Brand-specific variations of tobacco ingredients and mixtures markedly influence PM emissions under ventilation conditions. The most efficient ventilation mode to reduce PM exposure was achieved by opening the passenger´s window 10 cm and turning the onboard ventilation on power level 2/4. In-vehicle smoking should be banned to preserve innocent risk groups (e.g., children) from harm.

## Introduction

Despite years of subversive manipulations by the tobacco industry, many governments began to recognize the health threat caused by passive tobacco exposure in the past decades and passed legislation (e.g., banning smoke from public places, increasing taxes, prohibiting tobacco advertisement, integrating graphic warnings on packages)^1,2^. Nonetheless, the prevalence of tobacco consumption was only reduced by an estimated 10.4% since the beginning of the twenty-first century. Therefore, approximately 20.4% of the world’s population (aged ≥ 15) was still consuming tobacco products in 2020^3^. The global tobacco epidemic results in about eight million deaths per year, representing the most preventable cause of death worldwide^4^. Potentially deadly diseases, including cancer, cardiovascular, and pulmonary diseases^5,6^ can be linked to the hazardous smoke generated by cigarette combustion. The smoke contains over 5000 chemicals, many are known to be toxic^7–9^.

Also, non-smokers can be harmed by second-hand smoke (SHS), accounting for approximately 1.2 million deaths per year^4,10^. Children represent a group of particularly vulnerable individuals not able to defend themselves from second hand smoke (SHS) exposure. Many diseases during childhood can be linked to SHS (e.g., asthma, leukemia, bronchitis, otitis media, sudden infant death syndrome, etc.)^11,12^. Even prenatal tobacco smoke exposure can already harm the infant through the induction of different molecular and genetic mechanisms leading to poor birth outcomes and fetal maldevelopment^12^. In addition, infants take up high doses of particles per kg bodyweight from SHS. Fine and ultra-fine particles, in particular, are deposited in high concentrations in the alveolar and head regions of the infant and can lead to various avoidable diseases^13^.

Measurements of particulate matter (PM) emissions from cigarette smoke can aid in determining their danger to human health. PM is differentiated according to its size into PM_10_ (particles ≤ 10 µm ), PM_2.5_ (particles ≤ 2.5 µm), and PM_1_ (particles ≤ 1 µm)^14^_._ In 2021, the WHO published its updated air quality guidelines. They recommended a maximum 24-h PM_10_ of 45 µg/m^3^, while PM_2.5_ should not exceed 15 µg/m^3^ ^15^.

The development of an automatic environmental tobacco smoke emitter (AETSE) has enabled recent investigators to measure the PM emissions of tobacco smoke without being exposed to the harmful SHS. So far, this new technique has mainly been used under indoor conditions^16^. Therefore, it is of tremendous interest to investigate PM accumulations under in-vehicle conditions. Utilizing the Mitsubishi Space Runner with its integrated AETSE and cigarette smoking device from the TAPaC study presents the ideal platform for this research, which would otherwise have been unethical due to the exposure to tobacco smoke^17^.

When smoking in a confined space such as a car, smokers usually try to prevent too high concentrations of smoke due to ocular discomfort with irritation of the conjunctiva. However, precise knowledge on how to reduce smoke concentrations is not available and drivers either tend to open windows or activate ventilation or combine both measures.

We here aimed to investigate the effect of 7 different ventilation scenarios on the PM concentration in a driver´s cabin by comparing PM emissions of three tobacco products with varying ingredients and mixtures under simulated driving conditions.

## Methods

### Experimental setup

The methodological setup has been described earlier in the TAPaC study by Pitten et al.^17^. In brief, a compact car (Mitsubishi Space Runner 1991–1999) was stationed in a garage and equipped with an automatic environmental tobacco smoke emitter (AETSE) and a laser aerosol spectrometer (LAS) Grimm Model 11 R. Just before the measurements started, the LAS was calibrated by Grimm Group in November 2021. The car itself has a total interior volume of 3.709 m^3^ ^18^. The AETSE consists of a plunger inside a 200 ml glass syringe. As the plunger moves back, a negative pressure is created, and the syringe is filled with smoke. The smoke is expelled through the forward movement of the plunger, thus imitating a smoking process^16^. The AETSE was positioned behind the driver's seat and connected via a polyamide tube with the cigarette smoking device which was located on the passenger´s seat. The expelled smoke from the AETSE was transferred via a polyamide tube and released by a valve located at the head region of the passenger seat. Consequently, the experimental design allowes to imitate a smoker sitting on the passenger seat.

Two remotely controllable fans (Model: Master BML 4800) were installed inside the car cabin to quickly remove the harmful tobacco smoke from the car and garage. Two outer-placed fans (Model: TTV 4500 HP) were installed on both sides of the front window (driver´s side and passenger´s side) at 113 cm height. They were positioned 20 cm in front of the window edges and did not overlap with the hood or front screen of the car. The fans can imitate an airflow capable of mimicking a vehicle during its motion. For this study, only the fan on the smoker´s side was used and turned on at the highest power level, creating an airstream of 25.1 to 25.8 km/h measured at a distance of 5 cm with a handheld anemometer (Voltcraft PL-130 AN). The airstream speed at a distance of one meter was 15.9 to 17.4 km/h.

### Tobacco products

3R4F reference cigarettes (3R4F)^19^, Marlboro red (MR), and Marlboro gold (MG) were used (Table 1). Marlboro cigarettes are products of Phillip Morris International^20^.

**Table 1:** Content of cigarettes ^18,20,21^.

	3R4F reference (mg)	Marlboro red (mg)	Marlboro gold (mg)
Tar	9.4	10	6
Nicotine	0.73	0.8	0.5
Carbon monoxide	12	10	7

### Smoking protocol

A single cigarette at a time was remotely ignited. After cigarette ignition, two initiation puffs were taken with a time interval of 1 s (s). Over the course of 4.5 min in total, additional 8 puffs were taken (1 puff every 30 s). Each puff lasted for 3 s and had a volume of 40 ml. After 10 puffs, the cigarette was automatically expelled and extinguished in a water bath.

Before igniting the next cigarette, the car cabin was ventilated for at least 5 min using the integrated fans. The researcher could open the tailgate of the car, step out of the garage, and remotely turn on the fans.

### Measuring system

The LAS measured the PM concentration every 6 s through a mobile tube placed on the driver’s seat. The mobile tube was positioned 70 cm next to the burning cigarette. The passenger´s window was 105 cm beside the mobile tube, while the driver´s window was located at a distance of 65 cm. The LAS can measure particles from 0.25 to 32 µm^17^. It automatically differentiates particles according to their size into categories PM_10_, PM_2.5_, and PM_1_. Positioning a specialized sensor next to the mobile tube enabled the operator to acquire exact temperature and relative humidity (RH) data during the PM measurements ^17^.

### Measuring conditions

Measurements were conducted under seven different ventilation conditions. For all investigated conditions (except condition no. 1) applied: The onboard ventilation was turned on power level 2/4. Fresh air entered the vehicle through a large duct at the front of the car and was directed towards the windshield by the onboard ventilation system. The recirculation mode was not in use. Only the passenger side window was opened. The outer-placed fan on the passenger side window has three power levels. Only the highest power level (3/3) was used (15.9–17.4 km/h at one meter distance) under C3, C5, and C7 to simulate the airflow of a driving car.

- C1: Windows closed, car ventilation turned off, outside fan turned off.
- C2: Window 10 cm opened.
- C3: Window 10 cm opened with the outside fan turned on at highest power level (3/3).
- C4: Window half-opened.
- C5: Window half-opened with the outside fan turned on at highest power level (3/3).
- C6: Window fully opened.
- C7: Window fully opened with the outside fan turned on at highest power level (3/3).

### Data processing and analysis

PM measurements were divided into three phases:

- 1: PM measurement started after the vehicle cabin has been ventilated for at least 5 min.
- 2: PM was measured between cigarette ignition and extinguishment (4.5 min).
- 3: PM was measured for 5.5 additional min after the cigarette had been extinguished.

The statistics software GraphPad Prism version 6 (GraphPad Software, La Jolla, California, USA) was used to evaluate and compare the generated data of all 7 test conditions. To check Gaussian distribution, the following tests were performed: Shapiro–Wilk, D`Agostino-Pearson, and Kolmogorov–Smirnov test (passed). One-way analysis of variance (ANOVA) with Tukey’s multiple comparison test was used to analyze the level of significance (*p* = 0.05). The mean concentrations (C_mean_) of PM after 4.5 min and 10 min were evaluated from data sets containing 101 measurements per cigarette. For each ventilation condition, 24 cigarettes were smoked, therefore, each condition was replicated 24 times. The same data sets were also used to analyze peak emissions of PM at exactly 4.5 min (46th measurement) and 10 min (101st measurement). The baseline PM exposure before cigarette ignition was calculated using the time interval between the end of the 3rd phase and beginning of the 1st phase.

## Results

Before cigarette ignition, PM_10_, PM_2.5_, and PM_1_ accounted for an average baseline PM exposure of 16.6 ± 3.4 µg/m^3^, 15.1 ± 3.4 µg/m^3^, and 14.1 ± 3.6 µg/m^3^, respectively.

Tables 2 and 3 show the mean concentrations of PM_10_, PM_2.5_, and PM_1_ values of all tested tobacco products after 4.5 and 10 min, respectively. C_mean_ of PM_10_ after 4.5 min was between 87.6 to 1218 µg/m^3^, while PM_10_ after 10 min was between 68,7 and 1697 µg/m^3^. C_mean_ of PM_2.5_ after 4.5 and 10 min ranged from 86.8 to 1198 µg/m^3^ and from 68.2 to 1659 µg/m^3^, respectively. C_mean_ of PM_1_ after 4.5 min was between 84.3 and 959.7 µg/m^3^, while PM_1_ after 10 min ranged from 66.1 to 1263 µg/m^3^. Additionally, Tables 2 and 3 contain data about the PM peaks at 4.5 and 10 min. Peaks are the average of single measurement values at a given time per condition. Peaks of PM_10_ at 4.5 min were between 106.5 and 2261 µg/m^3^, while PM_10_ peaks at 10 min were between 14.6 and 2185 µg/m^3^. Peaks of PM_2.5_ at 4.5 and 10 min ranged from 106.3 to 2217 µg/m^3^ and from 14.5 to 2146 µg/m^3^, respectively. PM_1_ peaks at 4.5 min were between 103 and 1442 µg/m^3^, while peaks at 10 min ranged from 13.5 to 1421 µg/m^3^. The percentage changes between measured PM concentrations are found as Supplementary Table S1 online. Only same tobacco products are compared (e.g., MR with MR) under different ventilation conditions (C1–C7). Tobacco products smoked under C1 showed drastically increased PM concentrations compared to C2 to C7 (*p* < 0.0001). For example, PM_2.5_ concentrations of MR under C7 after 4.5 and 10 min were 99.4 and 82.1 µg/m^3^, respectively. On the contrary, PM_2.5_ of MR under C1 after 4.5 and 10 min was 908.7 to 1488 µg/m^3^, respectively. After extinguishing the cigarette, PM mean values continued to rise under C1 (3R4F: 31.6–39.3%, MR: 43.1–64.2%, MG: 38.3–48.1%) because PM concentrations decrease slower under conditions without ventilation compared to conditions with ventilation. PM C_mean_ of C2 to C7 decreased after 10 min compared to 4.5 min (3R4F: − 15.2 to − 28.6%, MR: − 12.3 to − 27.8%, MG: − 16.3 to − 27.5%). PM mean values of ventilated conditions (C2–C7) after 4.5 and 10 min were more than 90% lower than under C1 (see Supplementary Table S1 online). The lowest PM concentrations were measured under C2 (Tables 2, 3). PM_2.5_ C_mean_ of MG under C2 after 4.5 and 10 min was 106.1 and 82.6 µg/m^3^, respectively, while PM_2.5_ peaks at 4.5 and 10 min, respectively, accounted for 106.3 and 23.7 µg/m^3^.

**Table 2:** Mean concentrations of PM_10_, PM_2.5_, and PM_1_ after 4.5 min, and peak emissions at 4.5 min.

Condition	Tobacco products	C_mean_ PM_10_ [µg/m^3^]	C_mean_ PM_2.5_ [µg/m^3^]	C_mean_ PM_1_ [µg/m^3^]
C1	3R4F	A: 1218 ± 299.9	A: 1198 ± 296.5	A: 959.7 ± 283.6
		B: 2261 ± 396.8	B: 2217 ± 395.2	B: 1442 ± 201
C1	MR	A: 920.8 ± 173.3	A: 908.7 ± 169.5	A: 751.3 ± 105.8
		B: 1415 ± 252	B: 1396 ± 245.6	B: 1076 ± 138.7
C1	MG	A: 859.8 ± 259.3	A: 846.1 ± 252.9	A: 696.8 ± 165.4
		B: 1601 ± 546.3	B: 1576 ± 530.9	B: 1192 ± 271.8
C2	3R4F	A: 108.7 ± 16.5	A: 108.1 ± 16.4	A: 104.9 ± 15.6
		B: 144.1 ± 32.2	B: 143.7 ± 32.1	B: 137.4 ± 29.5
C2	MR	A: 107.8 ± 18.7	A: 106.1 ± 18.2	A: 101.9 ± 16.8
		B: 147.6 ± 29.6	B: 146.7 ± 29.1	B: 139.1 ± 26.2
C2	MG	A: 87.6 ± 13.6	A: 86.8 ± 13.5	A: 84.3 ± 12.7
		B: 106.5 ± 16	B: 106.3 ± 15.8	B: 103 ± 14.8
C3	3R4F	A: 164.2 ± 12.6	A: 162.7 ± 13	A: 156.3 ± 12.5
		B: 234.9 ± 20.9	B: 233.6 ± 20.5	B: 221.1 ± 18.1
C3	MR	A: 153 ± 9.6	A: 150 ± 9.1	A: 142.4 ± 8.4
		B: 235.4 ± 20.5	B: 231.1 ± 18.7	B: 214.9 ± 16
C3	MG	A: 119.1 ± 13.7	A: 117.9 ± 14	A: 114.1 ± 12.9
		B: 170.8 ± 21	B: 170 ± 20.8	B: 163.2 ± 18.9
C4	3R4F	A: 123.4 ± 16.3	A: 122.7 ± 16.2	A: 118.5 ± 15.6
		B: 165.2 ± 42.5	B: 164.5 ± 42.4	B: 157.7 ± 39.2
C4	MR	A: 121.2 ± 21.1	A: 120.5 ± 20.8	A: 115.7 ± 18.5
		B: 155.8 ± 37	B: 155.1 ± 36.5	B: 147.7 ± 32.4
C4	MG	A: 109.6 ± 16.4	A: 109.3 ± 16.3	A: 106.5 ± 15.5
		B: 152.5 ± 31.6	B: 152.1 ± 31.6	B: 146.9 ± 29.6
C5	3R4F	A: 152.7 ± 11.2	A: 150.9 ± 11.2	A: 145.1 ± 10.3
		B: 205.7 ± 20.8	B: 204.2 ± 20.7	B: 193.1 ± 18.3
C5	MR	A: 136.4 ± 12.3	A: 134.4 ± 12.3	A: 128.8 ± 11.4
		B: 192.4 ± 21.6	B: 190.6 ± 21.1	B: 180.1 ± 19.1
C5	MG	A: 118.1 ± 11.5	A: 116.3 ± 11.5	A: 112.5 ± 11.1
		B: 161.3 ± 19.2	B: 159.3 ± 19.2	B: 152.5 ± 17.9
C6	3R4F	A: 268.4 ± 24.5	A: 266.2 ± 23.7	A: 251.2 ± 19.7
		B: 340.4 ± 61.5	B: 338.6 ± 60.9	B: 314.6 ± 52.5
C6	MR	A: 219.8 ± 33.4	A: 218.9 ± 33.2	A: 209.1 ± 30.1
		B: 259.7 ± 42.7	B: 258.5 ± 42.6	B: 244.9 ± 38.3
C6	MG	A: 213.2 ± 21.9	A: 212.4 ± 21.6	A: 204.3 ± 19.4
		B: 258 ± 28.4	B: 256.3 ± 28.5	B: 244.8 ± 25.9
C7	3R4F	A: 129.9 ± 18	A: 127.4 ± 17.7	A: 122.3 ± 16.5
		B: 168 ± 27.1	B: 165.2 ± 26.5	B: 157 ± 24.1
C7	MR	A: 100.9 ± 10.6	A: 99.4 ± 10.2	A: 95.6 ± 9.6
		B: 149.2 ± 9.7	B: 147.6 ± 9	B: 140.6 ± 8.1
C7	MG	A: 95.8 ± 16	A: 93.8 ± 15.9	A: 90.1 ± 14.8
		B: 127.7 ± 24.8	B: 126.2 ± 24.6	B: 120.8 ± 22.6

3R4F, 3R4F reference cigarette; MR, Marlboro red; MG, Marlboro gold; A, Mean concentration after a specific time interval; B, Peak emissions at a specific time; PM, Particulate matter; C, Condition; C1, Windows closed, car ventilation off, outside fan off; C2, Window 10 cm opened, car ventilation on; C3, Window 10 cm opened, car ventilation on, outside fan turned on at highest power level; C4, Window half-opened, car ventilation on; C5, Window half-opened, car ventilation on, outside fan turned on at highest power level; C6, Window fully opened, car ventilation on; C7, Window fully opened, car ventilation on, outside fan turned on at highest power level.

**Table 3:** Mean concentrations of PM_10_, PM_2.5_, and PM_1_ after 10 min, and peak emissions at 10 min.

Condition	Tobacco products	C_mean_ PM_10_ [µg/m^3^]	C_mean_ PM_2.5_ [µg/m^3^]	C_mean_ PM_1_ [µg/m^3^]
C1	3R4F	A: 1697 ± 386.7	A: 1659 ± 374.3	A: 1263 ± 260.1
		B: 2016 ± 121.7	B: 1971 ± 119.7	B: 1409 ± 85.4
C1	MR	A: 1512 ± 232.6	A: 1488 ± 225.8	A: 1075 ± 108.9
		B: 2185 ± 348.2	B: 2146 ± 336.7	B: 1421 ± 137.8
C1	MG	A: 1272 ± 217.7	A: 1253 ± 211.4	A: 964 ± 116.2
		B: 1641 ± 275.7	B: 1617 ± 268.2	B: 1190 ± 130.3
C2	3R4F	A: 79.7 ± 10.5	A: 79.3 ± 10.5	A: 76.8 ± 9.9
		B: 19.1 ± 3.5	B: 19 ± 3.5	B: 18.4 ± 3.5
C2	MR	A: 83.6 ± 13.6	A: 82.6 ± 13.2	A: 78.7 ± 12
		B: 20.4 ± 4.6	B: 20 ± 4.4	B: 19 ± 4.2
C2	MG	A: 68.7 ± 8	A: 68.2 ± 7.9	A: 66.1 ± 7.4
		B: 23.8 ± 1.7	B: 23.7 ± 1.6	B: 23.1 ± 1.5
C3	3R4F	A: 139 ± 6.6	A: 137.9 ± 6.5	A: 131.9 ± 6.1
		B: 42 ± 3.9	B: 41.4 ± 3.3	B: 40.1 ± 2.9
C3	MR	A: 134.1 ± 8.5	A: 131.6 ± 8.4	A: 123.9 ± 7.8
		B: 39.4 ± 3.8	B: 37.8 ± 3.5	B: 35.5 ± 3.1
C3	MG	A: 99.6 ± 8.9	A: 98.7 ± 9	A: 95.1 ± 8.5
		B: 28.4 ± 4.5	B: 27.9 ± 4.4	B: 26.9 ± 4.4
C4	3R4F	A: 88.5 ± 13	A: 87.9 ± 12.9	A: 84.6 ± 12.5
		B: 14.6 ± 3.2	B: 14.5 ± 3.1	B: 13.5 ± 3.2
C4	MR	A: 93.5 ± 18	A: 93 ± 17.8	A: 88.9 ± 16
		B: 19.6 ± 3.2	B: 19.5 ± 3.2	B: 18.7 ± 2.9
C4	MG	A: 83.9 ± 12.1	A: 83.6 ± 12.1	A: 81.1 ± 11.3
		B: 17.9 ± 2	B: 17.8 ± 2	B: 17.5 ± 1.9
C5	3R4F	A: 120.1 ± 8.1	A: 118.7 ± 8.1	A: 113.8 ± 7.5
		B: 38.8 ± 4.7	B: 38.24 ± 4.7	B: 37 ± 4.4
C5	MR	A: 109.9 ± 8.8	A: 108.2 ± 8.9	A: 103.1 ± 8.2
		B: 37.55 ± 4.1	B: 36 ± 3.5	B: 34.2 ± 3.5
C5	MG	A: 94.3 ± 5.8	A: 92.8 ± 5.7	A: 89.5 ± 5.2
		B: 35.6 ± 3	B: 34.7 ± 2.6	B: 33.5 ± 2.4
C6	3R4F	A: 196.2 ± 23.4	A: 194.7 ± 22.7	A: 183.8 ± 19.8
		B: 34.2 ± 4.3	B: 34.1 ± 4.4	B: 33 ± 4.6
C6	MR	A: 159 ± 23.3	A: 158.3 ± 23.2	A: 150.9 ± 21.3
		B: 25.3 ± 4.6	B: 25.16 ± 4.5	B: 24.4 ± 4.3
C6	MG	A: 155.2 ± 15.5	A: 154.4 ± 15.3	A: 148.1 ± 13.9
		B: 21.1 ± 3.8	B: 21 ± 3.7	B: 20.1 ± 3.5
C7	3R4F	A: 99.4 ± 13.5	A: 97.5 ± 13.3	A: 93.3 ± 12.5
		B: 27.4 ± 4.2	B: 26.8 ± 3.9	B: 25.3 ± 3.7
C7	MR	A: 83.6 ± 7.5	A: 82.1 ± 7	A: 78.5 ± 6.5
		B: 24.5 ± 4.5	B: 23.2 ± 3.4	B: 21.9 ± 3.2
C7	MG	A: 73.7 ± 11	A: 72 ± 11.2	A: 68.9 ± 10.5
		B: 20.2 ± 3.7	B: 19 ± 3.2	B: 17.9 ± 3.1

3R4F, 3R4F reference cigarette; MR, Marlboro red; MG, Marlboro gold; A, Mean concentration after a specific time interval; B, Peak emissions at a specific time; PM, Particulate matter; C, Condition; C1, Windows closed, car ventilation off, outside fan off; C2, Window 10 cm opened, car ventilation on; C3, Window 10 cm opened, car ventilation on, outside fan turned on at highest power level; C4, Window half-opened, car ventilation on; C5, Window half-opened, car ventilation on, outside fan turned on at highest power level; C6, Window fully opened, car ventilation on; C7, Window fully opened, car ventilation on, outside fan turned on at highest power level.

After 4.5 and 10 min, C2 to C7 showed no significant differences (*p* > 0.05) among each other in most cases. All exceptions can be found as Supplementary Table S2 online.

PM peaks at 4.5 and 10 min gained extremely high levels for all tested tobacco products under C1. PM peaks of C2 to C7 at 4.5 and 10 min were 77.2 to 99.3% lower than under C1. C1 showed significant differences (*p* < 0.0001) compared to C2 to C7 at 4.5 and 10 min. All PM peaks at 4.5 and 10 min with significant differences are listed as Supplementary Table S2 online.

**Figure 1:**
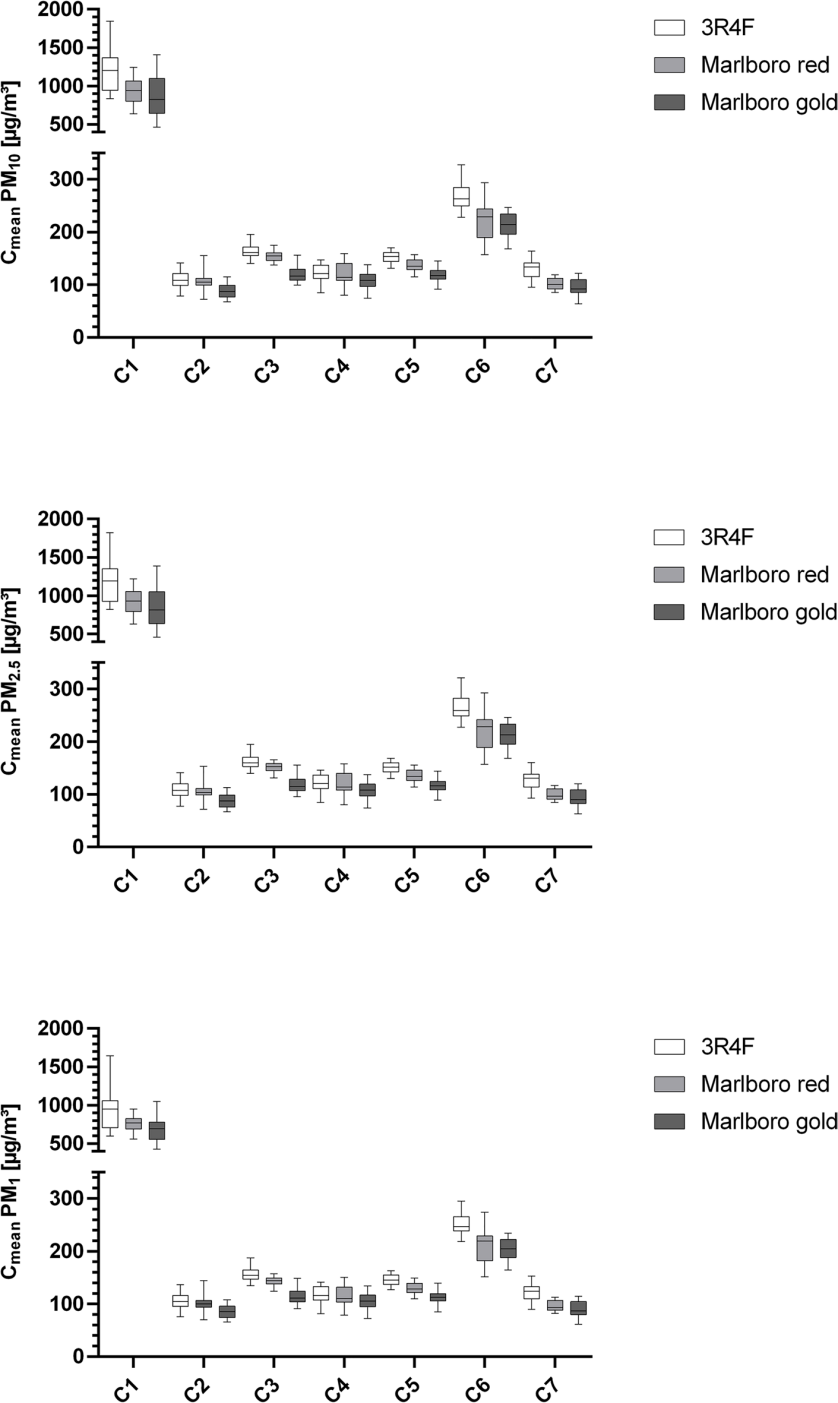
Boxplots (min to max whiskers) graphically display data of PM_10_, PM_2.5_, and PM_1_ concentrations after 4.5 min. PM, Particulate matter, 3R4F, 3R4F reference cigarette; C, Condition; C1, Windows closed, car ventilation off, outside fan off; C2, Window 10 cm opened, car ventilation on; C3, Window 10 cm opened, car ventilation on, outside fan turned on at highest power level; C4, Window half-opened, car ventilation on; C5, Window half-opened, car ventilation on, outside fan turned on at highest power level; C6, Window fully opened, car ventilation on; C7, Window fully opened, car ventilation on, outside fan turned on at highest power level.

**Figure 2:**
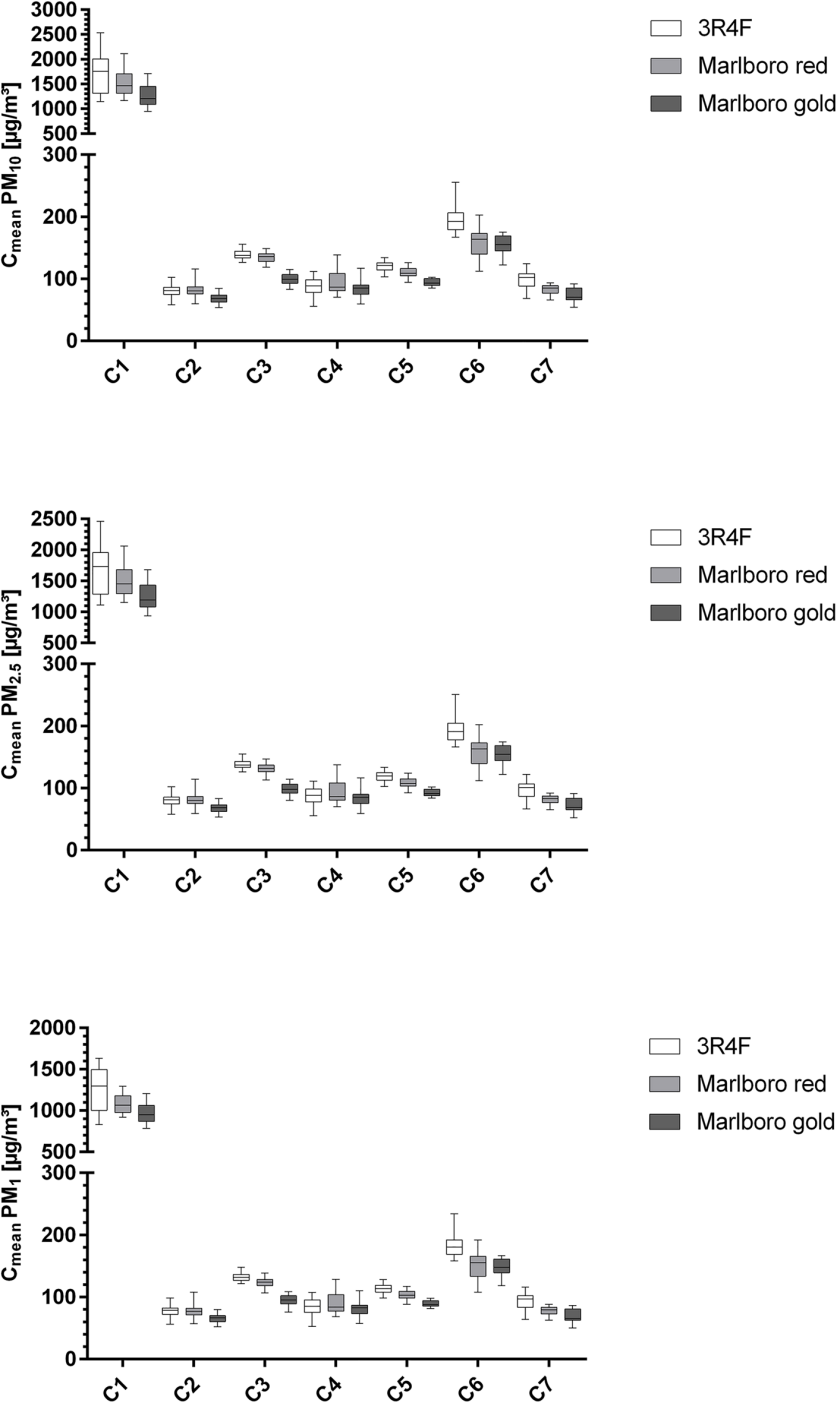
Boxplots (min to max whiskers) graphically display data of PM_10_, PM_2.5_, and PM_1_ concentrations after 10 min. PM, Particulate matter, 3R4F, 3R4F reference cigarette, C, Condition; C1, Windows closed, car ventilation off, outside fan off; C2, Window 10 cm opened, car ventilation on; C3, Window 10 cm opened, car ventilation on, outside fan turned on at highest power level; C4, Window half-opened, car ventilation on; C5, Window half-opened, car ventilation on, outside fan turned on at highest power level; C6, Window fully opened, car ventilation on; C7, Window fully opened, car ventilation on, outside fan turned on at highest power level.

Figures 1 and 2 illustrate the different PM emissions after 4.5 and 10 min, respectively, at conditions C1 to C7.

**Figure 3:**
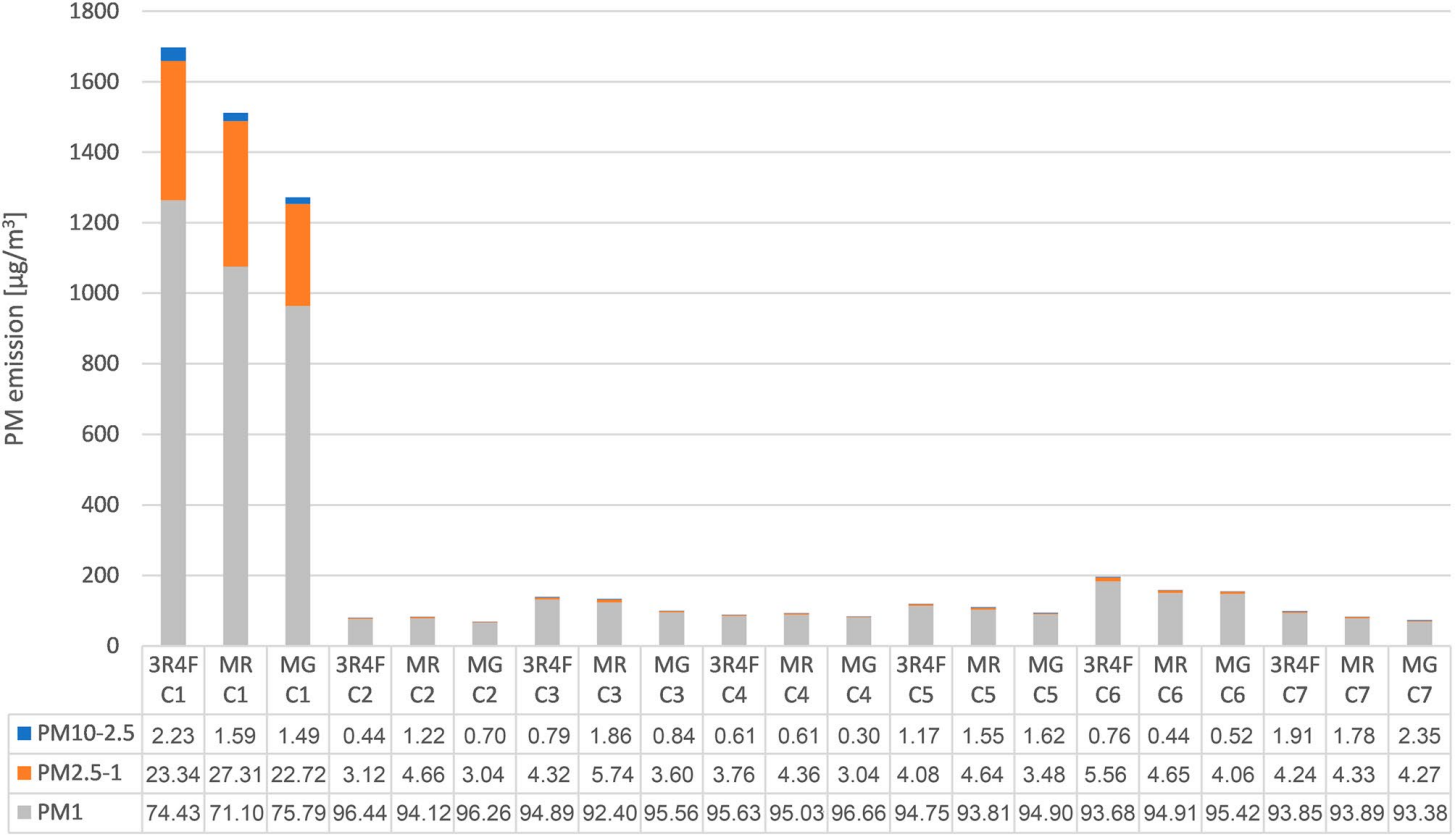
Distribution pattern of the mean concentrations (C_mean_) after 10 min under different ventilation conditions. Percentage information of the PM fractions PM_10−2.5_, PM_2.5−1_, and PM_1_ are rounded to two decimal places. PM, Particulate matter; 3R4F, 3R4F reference cigarette; MR, Marlboro red; MG, Marlboro gold; C, Condition; C1, Windows closed, car ventilation off, outside fan off; C2, Window 10 cm opened, car ventilation on; C3, Window 10 cm opened, car ventilation on, outside fan turned on at highest power level; C4, Window half-opened, car ventilation on; C5, Window half-opened, car ventilation on, outside fan turned on at highest power level; C6, Window fully opened, car ventilation on; C7, Window fully opened, car ventilation on, outside fan turned on at highest power level.

The distribution patterns of different particle size fractions for each condition (C1–C7) are displayed in Fig. 3. The lowest portion of PM_1_ (71–76%) had C1, while no other condition had higher values of PM_2.5−1_ (23–27%). PM_1_ for C2 to C7 ranged from 92 to 96%, while PM_2.5−1_ accounted for 3 to 6%. C1 to C7 showed low levels of PM_10−2.5_, ranging from 0.4 to 2.4%.

During the experiments, the measured relative air humidity was between 33 and 52%, while the temperature was between 12.6 and 16.7 °C.

## Discussion

Airborne PM poses high risks to human health^23^. Cigarettes produce toxic PM emissions, thereby harming not only the smoker but also surrounding individuals. Especially, smoking in an enclosed space, such as a car cabin, increase PM concentrations substantially. Frequently, children and adolescents become innocent victims of toxic SHS^24^. Narbi-Burza et al. conducted a study in the USA investigating parental smoke-free policies in cars. The study showed that 48% of smoking parents routinely expose their children to SHS in vehicles^25^.

The experimental setup and design allowed us to conduct this study entirely remote from a safe distance of at least five meters avoiding exposition to toxic SHS. Thanks to the unique and ethically acceptable measuring platform, the researcher could conduct the investigations without exposing himself or others to SHS.

Previous studies have investigated the effect of ventilation conditions on PM concentration inside a car cabin using human smokers^25–31^. Although it guaranteed a simple and realistic research setup, it also exposed at least the smoker to SHS. Furthermore, the puff volume of human smokers varies and depends on the smokers’ lung volume and smoking habits. That affects the exhaled PM emissions^33^. The AETSE of our experimental setup used equal puff volumes that eliminate the influence of human smoking behavior on PM exposure^16,34^. This study limitation can also be considered as advantage as standardized and comparable data is generated independently from the individual human smoker. Another technical limitation of the study was that particles of size < 0.25 µm could not be detected by the laser aerosol spectrometer (LAS) Grimm Model 11 R. Therefore, a small portion of fine particles (< 1 µm) and ultra-fine particles (< 0.1 µm) is not included in the data. Nevertheless, the majority of emitted PM_1_ is included in the analysis as the mass median diameter of mainstream cigarette smoke is 0.38 µm^35^. Since particularly ultra-fine particles are the source of many adverse health effects it would be useful to expand the technical equipment of the TAPaC measuring platform^36^.

In general, the study systematically focused on seven different ventilation conditions, opening no more than one window (passenger’s side) at once. Stop and go during real driving generates an inconstant and variable airstream that might result in incomparable PM data. Therefore, one powerful ventilator was used to simulate a constant and reproducible airstream. The setup in a garage allowed us to eliminate all exterior wind drifts that could otherwise have affected our investigations. Moreover, the garage had the advantage of small fluctuations in temperature.

PM concentrations under test condition C1 (windows closed, car ventilation off, fan off) remained extremely high, even after the cigarette was extinguished. The unventilated condition of C1 did not allow PM to escape from the vehicle, resulting in sustained high PM values. PM_2.5_ of Marlboro Gold under C1 after 10 min was 13.5% lower than Marlboro Red. Braun et al. showed 36% lower PM_2.5_ values of Marlboro Gold compared to Marlboro Red in an enclosed space of 2.88 m^3^ ^37^. This difference is most probably caused by the larger interior volume of the car (3.709 m^3^). The over 90-fold increase of PM_10_, PM_2.5_, and PM_1_ under C1 compared to baseline measurements was expectable, as previous studies have already shown extremely high concentrations of PM in confined spaces^37–39^. Measured PM concentrations of C2 to C7 compared to C1 were significantly lower (see Supplementary Table S2 online). The results are in line with a study conducted by Müller et al. that measured PM emissions of cigarettes smoked in a telephone booth. They showed 90% less PM_2.5_ under open-door ventilation in comparison to a closed telephone booth^40^. Our study came to comparable results after 10 min, showing ≥ 87.7% fewer PM_2.5_ at conditions C2 to C7 compared to C1 (see Supplementary Table S1 online).

Although different cigarette types were used, only a minority displayed significant differences under conditions with an opened window (C2 to C7) (see Supplementary Table S2 online). Nonetheless, different PM values can be seen comparing the reference cigarette, Marlboro Red, and Marlboro Gold under each test condition. With lower nicotine and tar content, Marlboro Gold presented with generally lower PM emissions than Marlboro Red and the reference cigarette (Figs. 1, 2). That is in line with Braun et al. showing that the PM emissions of cigarettes vary depending on their content and filter size^37^.

PM peaks at 4.5 and 10 min displayed similar percental changes as PM mean values (see Supplementary Table S1 online) because the cigarettes were extinguished after 4.5 min. Afterward, only PM of the remaining tobacco smoke was measured by the LAS. Thus, PM peaks were particularly high at 4.5 min and decreased markedly at 10 min measurements under C2 to C7.

PM_1_ accounts for the majority (71–97%) of tobacco smoke, while PM_2.5−1_ (3–27%) and PM_10−2.5_ (0.3–2.4%) account for the remaining parts (Fig. 3). Combustion of tobacco products generates primarily fine particulate matter (PM_2.5_)^41^. These extremely small particles are particularly dangerous for human health as they can be inhaled deeper into the lungs than coarse particles (PM_10-2.5_), thereby causing more damage^14^.

The variation of PM under conditions C2 to C7 is difficult to interpret and predict as many factors impact the interior PM concentrations. Ott et al. measured the air changes per hour in a car cabin. The study included multiple ventilation parameters (e.g., air conditioning, fan, speed, and degree of window opening) and showed that the air exchange per hour varies depending on the ventilation parameters^42^. Under real-life driving conditions, different speed levels affect the air inflow through the onboard ventilation system^43^. Future studies should install additional fans in front of the vehicle to investigate the impact of external airstream on PM elimination in a car cabin in more detail. In addition, a vehicle develops different interior and exterior pressure zones depending on the speed and other external factors. These pressure zones create variable air currents depending on how many windows are opened and to what degree^44^.

In our previous establishment of the presently used platform, we already found an effect of in-vehicle ventilation on PM with closed windows and showed that directing the ventilation towards the windshield can reduce PM by 67.4 to 74.4% compared to no ventilation^17^. Here we extend these findings and show that opening the window by different degrees and usage of the ventilators resulted in an even further reduction of PM (see Supplementary Table S1 online). Another example that demonstrates the effectiveness of combining in-vehicle ventilation with window opening can be seen by comparing a study published in 2012 with our results. The study was conducted by Northcross et al. and measured the PM_2.5_ emissions of three smoked cigarettes during 1 h inside a stationary vehicle. Although both front windows were opened during the investigations, the measured PM_2.5_ concentration (746.1 µg/m^3^) was more than three times higher compared to C6 in our study^30^. The main difference between the two experimental setups was that in our investigation, under C2 to C7, the onboard ventilation system was turned on, thereby increasing the air exchange rate^42^. One more research paper with a similar study design was conducted by Sohn et al. who measured PM_2.5_ of tobacco smoke inside a vehicle with three different window openings. PM_2.5_ was measured in the back seat while the driver smoked a single cigarette in a driving vehicle. The average PM_2.5_ concentrations during the smoking phase were substantially higher compared to C2 to C7 after 4.5 min in our study^28^. Nonetheless, both experiments exceeded the recommended WHO limits of PM by far^15^.

Although all cigarette types (3R4F, MR, MG) smoked under C1 presented with outstandingly high PM concentrations, the 3R4F reference cigarettes generated the highest emissions. PM_10_ after 10 min of cigarettes smoked under C1 was 38 times higher than the recommended WHO threshold, while PM_2.5_ exceeded the threshold by factor 111. The lowest concentration of PM was measured under C2. After 10 min, PM_10_ and PM_2.5_ exceeded the WHO threshold by factors 1.5 and 4.5, respectively. After 4.5 min, PM_10_ and PM_2.5_ were 1.9 and 5.8 times higher than the WHO threshold^15^ (Tables 2, 3). In summary, it is a given fact that under all conditions investigated in this study, dangerously high levels of PM from smoking cigarettes were present. Therefore, we recommend that regulators should focus on the role of tobacco smoking in cars.

## Conclusion

This study underlines the danger of smoking in cars. It disproves the belief that vehicle ventilation can protect non-smoking passengers from SHS and concomitant PM and provides information about PM emissions under different ventilation conditions. Nonetheless, a drastic decrease of PM concentrations could be detected after opening one window by different degrees. The lowest PM concentrations were measured with the passenger´s window 10 cm opened, onboard vehicle ventilation turned on power level 2/4 and the fan turned off. It is the result of multiple ventilation parameters (fan speed, onboard ventilation, external pressure zones, degree of window opening) influencing the car´s air exchange per hour. However, once the window was opened the different degrees of window opening did not majorly impact the PM concentrations inside the vehicle cabin. This data points to the urgent need for legislation on tobacco use in the presence of children and pregnant women in vehicles.

## Supplementary Information

Supplementary Legends.

Supplementary Table S1.

Supplementary Table S2.

## Data Availability

The datasets used and/or analyzed during the current study are available from the corresponding author upon reasonable request.

## Abbreviations

AETSE: Automatic environmental tobacco smoke emitter
ANOVA: Analysis of variance
C_mean_: Mean concentration
C1: Windows closed, car ventilation turned off, fan turned off
C2: Window 10 cm opened, car ventilation turned on, with the air directed towards the windshield
C3: Window 10 cm opened, car ventilation turned on, with the air directed towards the windshield, fan turned on
C4: Window half-opened, car ventilation turned on, with the air directed towards the windshield
C5: Window half-opened, car ventilation turned on, with the air directed towards the windshield, fan turned on
C6: Window fully opened, car ventilation turned on, with the air directed towards the windshield
C7: Window fully opened, car ventilation turned on, with the air directed towards the windshield, fan turned on
FCTC: Framework Convention on Tobacco Control
LAS: Laser aerosol spectrometer
MR: Marlboro red
MG: Marlboro gold
p: Probability value
PM: Particulate matter
s: Seconds
SHS: Second-hand smoke
ToPIQ: Tobacco smoke particles and indoor air quality
WHO: World Health Organization
3R4F: 3R4F reference cigarettes
