# Supplementary material for: Impact of different ventilation conditions on tobacco smoke-associated particulate matter emissions in a car cabin using the TAPaC platform
Source: Sci Rep. 2023 May 22;13:8216. doi: 10.1038/s41598-023-35208-2 (PMC10203320; doi:10.1038/s41598-023-35208-2)
Supplement: Supplementary file 1 — Supplementary Legends. [file 41598_2023_35208_MOESM1_ESM.docx]

**Caption of Tables S1 and S2**

**Table S1:** Percentage changes of C_mean_ and peak emissions of PM_10_, PM_2.5_, and PM_1_ (as listed in Table 2 and Table 3) between 4.5 and 10 min and between condition C1 and conditions C2 to C7.

3R4F: 3R4F reference cigarette. MR: Marlboro red. MG: Marlboro gold. PM: Particulate matter. C: Condition C1: Windows closed, car ventilation off, outside fan off. C2: Window 10 cm opened, car ventilation on. C3: Window 10 cm opened, car ventilation on, outside fan turned on at highest power level. C4: Window half-opened, car ventilation on. C5: Window half-opened, car ventilation on, outside fan turned on at highest power level; C6: Window fully opened, car ventilation on. C7: Window fully opened, car ventilation on, outside fan turned on at highest power level. A: Percentage change of PM between 4.5 and 10 min for each condition. B: Percentage change of PM between C1 and C2 to C7 after 4.5 min. C: Percentage change of PM between C1 and C2 to C7 after 10 min. D: Percentage change of PM peaks between 4.5 and 10 min for each condition. E: Percentage change of PM peaks between C1 and C2 to C7 at 4.5 min. F: Percentage change of PM peaks between C1 and C2 to C7 at 10 min.

**Table S2:** List of conditions compared with one another displaying significant differences (p < 0.05).

3R4F: 3R4F reference cigarette. MR: Marlboro red. MG: Marlboro gold. PM: Particulate matter. C: Condition. C1: Windows closed, car ventilation off, outside fan off. C2: Window 10 cm opened, car ventilation on. C3: Window 10 cm opened, car ventilation on, outside fan turned on at highest power level. C4: Window half-opened, car ventilation on. C5: Window half-opened, car ventilation on, outside fan turned on at highest power level; C6: Window fully opened, car ventilation on. C7: Window fully opened, car ventilation on, outside fan turned on at highest power level.
