# Supplementary material for: Impact of different ventilation conditions on tobacco smoke-associated particulate matter emissions in a car cabin using the TAPaC platform
Source: Sci Rep. 2023 May 22;13:8216. doi: 10.1038/s41598-023-35208-2 (PMC10203320; doi:10.1038/s41598-023-35208-2)
Supplement: Supplementary file 2 — Supplementary Table S1. [file 41598_2023_35208_MOESM2_ESM.docx]

**Table S1:** Percentage changes of C_mean_ and peak emissions of PM_10_, PM_2.5_, and PM_1_ (as listed in Table 2 and Table 3) between 4.5 and 10 min and between condition C1 and conditions C2 to C7.

| Condition | Tobacco Products | Group | PM_10_ | PM_2.5_ | PM_1_ |
| --- | --- | --- | --- | --- | --- |
| C1 | 3R4F | A | + 39.3% | + 38.5% | + 31.6% |
|  |  | D | − 10.8% | − 11.1% | − 2.3% |
| C1 | MR | A | + 64.2% | + 63.8% | + 43.1% |
|  |  | D | + 54.4% | + 53.7% | + 32.1% |
| C1 | MG | A | + 47.9% | + 48.1% | + 38.3% |
|  |  | D | + 2.5% | + 2.6% | − 0.2% |
| C2 | 3R4F | A | − 26.7% | − 26.6% | − 26.7% |
|  |  | B | − 91.1% | − 91% | − 89.1% |
|  |  | C | − 95.3% | − 95.2% | − 93.9% |
|  |  | D | − 86.7% | − 86.8% | − 86.6% |
|  |  | E | − 93.6% | − 93.5% | − 90.5% |
|  |  | F | − 92.9% | − 99% | − 98.7% |
| C2 | MR | A | − 22.4% | − 22.2% | − 22.8% |
|  |  | B | − 88.3% | − 88.3% | − 86.4% |
|  |  | C | − 94.5% | − 94.5% | − 92.7% |
|  |  | D | − 86.2% | − 86.4% | − 86.4% |
|  |  | E | − 89.6% | − 89.5% | − 87.1% |
|  |  | F | − 99% | − 99.1% | − 98.7% |
| C2 | MG | A | − 21.6% | − 21.5% | − 21.6% |
|  |  | B | − 89.8% | − 89.7% | − 87.9% |
|  |  | C | − 94.6% | − 94.6% | − 93.2% |
|  |  | D | − 77.6% | − 77.7% | − 77.6% |
|  |  | E | − 93.3% | − 93.3% | − 91.4% |
|  |  | F | − 98.5% | − 98.5% | − 98.1% |
| C3 | 3R4F | A | − 15.3% | − 15.2% | − 15.6% |
|  |  | B | − 86.5% | − 86.4% | − 83.7% |
|  |  | C | − 91.8% | − 91.7% | − 89.6% |
|  |  | D | − 82.1% | − 82.3% | − 81.9% |
|  |  | E | − 89.6% | − 89.5% | − 84.7% |
|  |  | F | − 97.9% | − 97.9% | − 97.2% |
| C3 | MR | A | − 12.4% | − 12.3% | − 13% |
|  |  | B | − 83.4% | − 83.5% | − 81% |
|  |  | C | − 91.1% | − 91.2% | − 88.5% |
|  |  | D | − 83.3% | − 83.6% | − 83.5% |
|  |  | E | − 83.4% | − 83.4% | − 80% |
|  |  | F | − 98.2% | − 98.2% | − 97.5% |
| C3 | MG | A | − 16.4% | − 16.3% | − 16.6% |
|  |  | B | − 86.1 | − 86.1% | − 83.6% |
|  |  | C | − 92.2% | − 92.1% | − 90.1% |
|  |  | D | − 83.4% | − 83.6% | − 83.5% |
|  |  | E | − 89.3% | − 89.2% | − 86.3% |
|  |  | F | − 98.3% | − 98.3% | − 97.7% |
| C4 | 3R4F | A | − 28.3% | − 28.3% | − 28.6% |
|  |  | B | − 89.9% | − 89.8% | − 87.7% |
|  |  | C | − 94.8% | − 94.7% | − 93.3% |
|  |  | D | − 91.2% | − 91.2% | − 91.4% |
|  |  | E | − 92.7% | − 92.6% | − 89.1% |
|  |  | F | − 99.3% | − 99.3% | − 99% |
| C4 | MR | A | − 22.8% | − 22.8% | − 23.2% |
|  |  | B | − 86.8% | − 86.7% | − 84.6% |
|  |  | C | − 93.8% | − 93.8% | − 91.7% |
|  |  | D | − 87.4% | − 87.5% | − 87.4% |
|  |  | E | − 89% | − 88.9% | − 86.3% |
|  |  | F | − 99.1% | − 99.1% | − 98.7% |
| C4 | MG | A | − 23.5% | − 23.5% | − 23.9% |
|  |  | B | − 87.3% | − 87.1% | − 84.7% |
|  |  | C | − 93.4% | − 93.3% | − 91.6% |
|  |  | D | − 88.3% | − 88.3% | − 88.1% |
|  |  | E | − 90.5% | − 90.3% | − 87.7% |
|  |  | F | − 98.9% | − 98.9% | − 98.5% |
| C5 | 3R4F | A | − 21.3% | − 21.3% | − 21.6% |
|  |  | B | − 87.5% | − 87.4% | − 84.9% |
|  |  | C | − 92.9% | − 92.9% | − 90% |
|  |  | D | − 81.1% | − 81.3% | − 80.9% |
|  |  | E | − 90.9% | − 90.8% | − 86.6% |
|  |  | F | − 98.1% | − 98.1% | − 97.4% |
| C5 | MR | A | − 19% | − 19.5% | − 20% |
|  |  | B | − 85.2% | − 85.2% | − 82.9% |
|  |  | C | − 92.7% | − 92.7% | − 90.4% |
|  |  | D | − 80.5% | − 81.1% | − 81% |
|  |  | E | − 86.4% | − 86.3% | − 83.3% |
|  |  | F | − 98.3% | − 98.3% | − 97.6% |
| C5 | MG | A | − 20.1% | − 20.2 | − 20.4% |
|  |  | B | − 86.3% | − 86.3% | − 83.9% |
|  |  | C | − 94.3% | − 92.6% | − 90.7% |
|  |  | D | − 78% | − 78.2% | − 78% |
|  |  | E | − 89.9% | − 89.9% | − 87.2% |
|  |  | F | − 97.8% | − 97.9% | − 97.2% |
| C6 | 3R4F | A | − 26.9% | − 26.9% | − 26.8% |
|  |  | B | − 78% | − 77.8% | − 73.8% |
|  |  | C | − 88.4% | − 88.3% | − 85.5% |
|  |  | D | − 90% | − 89.9% | − 89.5% |
|  |  | E | − 84.9% | − 84.7% | − 78.2% |
|  |  | F | − 98.3% | − 98.3% | − 97.7% |
| C6 | MR | A | − 27.7% | − 27.7% | − 27.8% |
|  |  | B | − 76.1% | − 75.9% | − 72.2% |
|  |  | C | − 89.5% | − 89.4% | − 85% |
|  |  | D | − 90.3% | − 90.3% | − 90% |
|  |  | E | − 81.6% | − 81.5% | − 77.2% |
|  |  | F | − 98.8% | − 98.8% | − 98.3% |
| C6 | MG | A | − 27.2% | − 27.3% | − 27.5% |
|  |  | B | − 75.2% | − 74.9% | − 70.7% |
|  |  | C | − 87.8% | − 87.7% | − 84.6% |
|  |  | D | − 91.8% | − 91.8% | − 91.8% |
|  |  | E | − 83.9% | − 83.7% | − 79.5% |
|  |  | F | − 98.7% | − 98.7% | − 98.3% |
| C7 | 3R4F | A | − 23.5% | − 23.5% | − 23.7% |
|  |  | B | − 89.3% | − 89.4% | − 87.3% |
|  |  | C | − 94.1% | − 94.1% | − 92.6% |
|  |  | D | − 83.7 | − 83.8% | − 83.9% |
|  |  | E | − 92.6% | − 92.5% | − 89.1% |
|  |  | F | − 98.6% | − 98.6% | − 98.2% |
| C7 | MR | A | − 17.2% | −17.4% | − 18% |
|  |  | B | − 89% | − 89.1% | − 87.3% |
|  |  | C | − 94.5% | − 94.5% | − 92.7% |
|  |  | D | − 83.6% | − 84.3% | − 84.4% |
|  |  | E | − 89.5% | − 89.4% | − 86.9% |
|  |  | F | − 98.9% | − 98.9% | − 98.5% |
| C7 | MG | A | − 23% | − 23.2% | − 23.6% |
|  |  | B | − 88.9% | − 88.9% | − 87.1% |
|  |  | C | − 94.2% | − 94.3% | − 92.9% |
|  |  | D | − 84.2% | − 84.9% | − 85.2% |
|  |  | E | − 92% | − 92% | − 89.9% |
|  |  | F | − 98.8% | − 98.7% | − 98.5% |

3R4F: 3R4F reference cigarette. MR: Marlboro red. MG: Marlboro gold. PM: Particulate matter. C: Condition C1: Windows closed, car ventilation off, outside fan off. C2: Window 10 cm opened, car ventilation on. C3: Window 10 cm opened, car ventilation on, outside fan turned on at highest power level. C4: Window half-opened, car ventilation on. C5: Window half-opened, car ventilation on, outside fan turned on at highest power level; C6: Window fully opened, car ventilation on. C7: Window fully opened, car ventilation on, outside fan turned on at highest power level. A: Percentage change of PM between 4.5 and 10 min for each condition. B: Percentage change of PM between C1 and C2 to C7 after 4.5 min. C: Percentage change of PM between C1 and C2 to C7 after 10 min. D: Percentage change of PM peaks between 4.5 and 10 min for each condition. E: Percentage change of PM peaks between C1 and C2 to C7 at 4.5 min. F: Percentage change of PM peaks between C1 and C2 to C7 at 10 min.
