# Supplementary material for: Impact of different ventilation conditions on tobacco smoke-associated particulate matter emissions in a car cabin using the TAPaC platform
Source: Sci Rep. 2023 May 22;13:8216. doi: 10.1038/s41598-023-35208-2 (PMC10203320; doi:10.1038/s41598-023-35208-2)
Supplement: Supplementary file 3 — Supplementary Table S2. [file 41598_2023_35208_MOESM3_ESM.docx]

**Table S2:** List of conditions compared with one another displaying significant differences (p < 0.05).

| Modes | PM_10_ | PM_2.5_ | PM_1_ |
| --- | --- | --- | --- |
| Mean values after 4.5 min | - C1 vs. C2 to C7: p < 0.0001 - 3R4F C2 vs. 3R4F C6: p < 0.0001 - MR C2 vs. MR C5: p = 0.0051 - MG C2 vs. MG C6: p = 0.0002 - 3R4F C4 vs. 3R4F C6: p < 0.0001 - MR C4 vs. MR C6: p = 0.038 - MG C4 vs. MG C6: p = 0.02 - 3R4F C5 vs. 3R4F C6: p = 0.0029 - 3R4F C6 vs. 3R4F C7: p < 0.0001 - MR C6 vs. MR C7: p = 0.0022 | - C1 vs. C2 to C7: p < 0.0001 - 3R4F C2 vs. 3R4F C6: p = 0.0046 - MR C2 vs. MR C6: p = 0.0034 - MG C2 vs. MG C6: p = 0.0004 - MG C3 vs. MG C6: p = 0.0452 - 3R4F C4 vs. 3R4F C6: p < 0.0001 - MR C4 vs. MR C6: p = 0.0313 - MG C5 vs. MG C6: p = 0.0367 - 3R4F C6 vs. 3R4F C7: p < 0.0001 - MR C6 vs. MR C7: p = 0.0014 - MG C6 vs. MG C7: p = 0.0013 | - C1 vs. C2 to C7: p < 0.0001 - 3R4F C2 vs. 3R4F C6: p < 0.0001 - MR C2 vs. MR C6: p = 0.0001 - MG C2 vs. MG C6: p < 0.0001 - 3R4F C3 vs. 3R4F C6: p = 0.0016 - MG C3 vs. MG C6: p = 0.0042 - 3R4F C4 vs. 3R4F C6: p < 0.0001 - MR C4 vs. MR C6: p = 0.0027 - MG C4 vs. MG C6: p = 0.0011 - 3R4F C5 vs. 3R4F C6: p = 0.0001 - MR C5 vs. MR C6: p = 0.0249 - MG C5 vs. MG C6: p = 0.0031 - 3R4F C6 vs. 3R4F C7: p < 0.0001 - MR C6 vs. MR C7: p < 0.0001 - MG C6 vs. MG C7: p < 0.0001 |
| Mean values after 10 min | - C1 vs. C2 to C7: p < 0.0001 - 3R4F C2 vs. 3R4F C6: p = 0.0293 | - C1 vs. C2 to C7: p < 0.0001 - 3R4F C2 vs. 3R4F C6: p = 0.022 | - C1 vs. C2 to C7: p < 0.0001 - 3R4F C2 vs. 3R4F C6: p = 0.0176 - MR C2 vs. MR C6: p = 0.0183 - MG C2 vs. MG C6: p = 0.0023 - 3R4F C4 vs. 3R4F C6: p < 0.0001 - MG C4 vs. MG C6: p = 0.0475 - 3R4F C5 vs. 3R4F C6: p = 0.0278 - 3R4F C6 vs. 3R4F C7: p = 0.0003 - MR C6 vs. MR C7: p = 0.0204 - MG C6 vs. MG C7: p = 0.0043 |
| Peaks at 4.5 min | - C1 vs. C2 to C7: p < 0.0001 - 3R4F C2 vs. 3R4F C6: p = 0.0058 - 3R4F C4 vs. 3R4F C6: p = 0.0231 - 3R4F C6 vs. 3R4F C7: p = 0.0327 | - C1 vs. C2 to C7: p < 0.0001 - 3R4F C4 vs. 3R4F C6: p = 0.0189 - 3R4F C6 vs. 3R4F C7: p = 0.0231 | - C1 vs. C2 to C7: p < 0.0001 - MR C2 vs. MR C6: p = 0.0029 - MG C2 vs. MG C6: p < 0.0001 - 3R4F C4 vs. 3R4F C6: p < 0.0001 - MR C4 vs. MR C6: p = 0.012 - MG C4 vs. MG C6: p = 0.013 - MG C5 vs. MG C6: p = 0.0292 - 3R4F C6 vs. 3R4F C7: p < 0.0001 - MR C6 vs. MR C7: p = 0.0127 - MG C6 vs. MG C7: p = 0.0001 |
| Peaks at 10 min | - C1 vs. C2 to C7: p < 0.0001 | - C1 vs. C2 to C7: p < 0.0001 | - C1 vs. C2 to C7: p < 0.0001 |

3R4F: 3R4F reference cigarette. MR: Marlboro red. MG: Marlboro gold. PM: Particulate matter.
C: Condition. C1: Windows closed, car ventilation off, outside fan off. C2: Window 10 cm opened, car ventilation on. C3: Window 10 cm opened, car ventilation on, outside fan turned on at highest power level. C4: Window half-opened, car ventilation on. C5: Window half-opened, car ventilation on, outside fan turned on at highest power level; C6: Window fully opened, car ventilation on. C7: Window fully opened, car ventilation on, outside fan turned on at highest power level.
